# Genome divergence between European anchovy ecotypes fuelled by structural variants originating from trans-equatorial admixture

**DOI:** 10.1098/rspb.2025.1416

**Published:** 2025-11-05

**Authors:** Laura Meyer, Pierre Barry, Alan Le Moan, Christine Arbiol, Rita Castilho, Carl Van der Lingen, Malika Chlaïda, Niall McKeown, Bruno Ernande, François Bonhomme, Pierre-Alexandre Gagnaire, Bruno Guinand

**Affiliations:** ^1^ ISEM, Univ Montpellier, CNRS, IRD, Montpellier, France; ^2^ Universidade do Porto Centro de Investigação em Biodiversidade e Recursos Genéticos, Vairão, Porto District, Portugal; ^3^ BIOPOLIS Program in Genomics, Biodiversity and Land Planning, CIBIO, Campus de Vairão, Vairão, Portugal; ^4^ Station Biologique de Roscoff, Roscoff, Brittany, France; ^5^ Universidade do Algarve Centro de Ciências do Mar, Faro, Faro District, Portugal; ^6^ Marine and Coastal Management, Department of Forestry, Fisheries and the Environment, Cape Town, South Africa; ^7^ University of the Western Cape, Cape Town, Western Cape, South Africa; ^8^ Research and Development Unit on Marine Biology, National Institute of Fisheries Research, Casablanca, Morocco; ^9^ Aberystwyth University, Aberystwyth, Wales, UK; ^10^ MARBEC, Montpellier, Occitanie, France

**Keywords:** ecotypes, structural variants, admixture, population genomics, evolutionary history

## Abstract

The formation of ecotypes is driven by evolutionary mechanisms that reduce gene flow through complex interactions among ecological, historical and genomic factors. In the European anchovy (*Engraulis encrasicolus*), marine and coastal ecotypes have been identified in the northeast Atlantic and the Mediterranean Sea, yet the genomic basis of their divergence remains unclear. Here, we present the first genome-scale analysis of this species complex, integrating whole-genome sequencing (WGS) and RAD-seq data from populations spanning its distribution range. In addition to the known marine and coastal ecotypes, we identify a previously undetected lineage extending from southern Morocco, through the Canary Islands, to South Africa. This southern Atlantic lineage exhibits a gradient of admixture with northern populations near the Atlantic–Mediterranean transition zone. Genomic differentiation landscapes reveal large regions of high linkage disequilibrium, probably corresponding to 13 structural variants (SVs) segregating within or between lineages. Notably, three of the six SVs contributing to the gene flow barrier between northern ecotypes originated in the southern lineage, supporting a partially shared evolutionary history between the coastal ecotype and the southern lineage. This study highlights how SVs that arose in geographically isolated lineages can act as key genetic elements in ecotype formation, reinforcing reproductive isolation through distinct evolutionary pathways.

## 1. Introduction

Speciation is a complex process shaped by temporal, spatial, ecological and genome architectural factors [1]. These factors influence diverse mechanisms of reproductive isolation (RI), contributing to the buildup of barriers to gene flow and the gradual strengthening of RI during speciation [2,3]. Although the multifaceted nature of speciation is widely recognized, early speciation genomics studies often focused on systems where divergence appears to be driven by one or a few dominant factors within a well-defined eco-evolutionary context. Ecotype formation is a prominent example, where genomic variation associated with distinct ecological habitats has traditionally been used to investigate ecologically driven speciation. In such cases, speciation is often assumed to be rapidly initiated by local adaptation, provided that divergent selection arises from habitat variation and sufficient genetic variation is available to selection [4,5]. However, recent advances in speciation genomics offer a more nuanced perspective, with growing evidence that ecotype formation results from the interplay of multiple factors [6,7].

A key insight from these genomic studies relates to the temporal dimension of ecotype formation, revealing that ecotype divergence often involves ancient variation. Even in cases where ecotypes were thought to arise due to recent habitat establishment, estimated divergence times frequently exceed those inferred from paleoenvironmental reconstructions or coalescent time distributions expected under panmixia [8–11]. This mismatch between ecological and genetic divergence time frames [6] may result from several mechanisms, including long-term balancing selection on the variants underlying ecotype divergence, ancestral population structure or admixture between divergent lineages.

Another recurrent finding is that genetic differences involved in ecotype formation tend to cluster in low-recombination regions, rather than being spread across the genome. These regions often contain structural variants (SVs), such as chromosomal inversions, which appear to play a major role in ecotype divergence [12–14]. Recent evidence suggests that SVs may form a substantial barrier to gene flow, maintaining at least moderate RI between lineages, particularly when they differ by multiple SVs [15]. Since recombination is largely suppressed in SVs like inversions, divergence can accumulate more readily in the absence of gene flow [16,17]. While SVs are often implicated in RI between ecotypes through their role in local adaptation by capturing multiple loci under environmental selection [18,19], they may also harbour coadapted gene complexes or Dobzhansky–Muller incompatibilities that also contribute to RI [20].

Understanding the origin of the genetic variation underlying ecotype divergence is a key step towards reconciling the multiple components of ecotype formation and identifying likely evolutionary scenarios. One such scenario, which accounts for both the temporal and ecological dimensions of ecotype formation, involves admixture between geographical lineages. Admixture is not merely a homogenizing process; it has also been linked to evolutionary diversification through several mechanisms. These include the transfer of locally adaptive variants via introgression [1], combinatorial speciation through the emergence of new allelic combinations favoured in different habitats [7] and the resolution of genomic incompatibilities between parental lineages [21]. In such scenarios, SVs may provide ‘pre-packaged’ divergent haplotypes that can readily contribute to RI following introgression [22,23] or may better resist re-homogenization compared to the collinear genome after the onset of gene flow [24,25]. However, further empirical evidence is required to clarify the role of admixture in driving ecotype divergence via the transfer of SVs.

Here, we investigate the role of SVs in ecotype formation within the European anchovy, *Engraulis encrasicolus sensu lato*. This species complex has formerly been shown to be subdivided into a marine ecotype (offshore and pelagic, *E. engraulis s. stricto*) and a coastal ecotype (nearshore, lagoonal and estuarine, *E. maeoticus*) that are able to co-exist in quasi-sympatry despite frequent hybridization [26]. Partial RI between these ecotypes is evidenced by both genetic and phenotypic differences (see [27] for a review) and their divergence involves a condensed genomic architecture, suggesting a potential role for SVs [26]. *E. encrasicolus* is distributed across the northeast Atlantic Ocean, Mediterranean Sea and Black Sea. Classically, its southern range limit along the western African coast is considered to be the south of the Gulf of Guinea, with *E. capensis* (southern African anchovy) described further south in the Benguela system off South Africa. However, some samples collected near the Atlantic–Mediterranean transition zone, off the Moroccan coast and near the Canary Islands, show genetic proximity with *E. capensis* [28,29], suggesting the presence of a third, previously unrecognized ancestry. These findings call for further investigation into potential admixture scenarios between *E. capensis* and the European anchovy lineages, and considering possible evolutionary impacts on the divergence between the marine and coastal ecotypes.

We present the first whole-genome sequencing (WGS) study of the *E. encrasicolus* species complex, offering a detailed characterization of its genetic structure and revealing the genomic architecture of divergence among anchovy lineages. To complement the WGS data, we incorporated RAD sequencing to characterise the eco-geographic structure of anchovy populations across a broad geographic range. This included individuals from both marine and coastal ecotypes in the northern part of the distribution, as well as anchovies from the Canary Islands, the Moroccan coast and South Africa. We aimed to determine whether and how these lineages have genetically interacted over their evolutionary history and to evaluate the potential role of SVs in driving their divergence.

## 2. Material and methods

### (a) Sampling and DNA extraction

**Figure 1.**
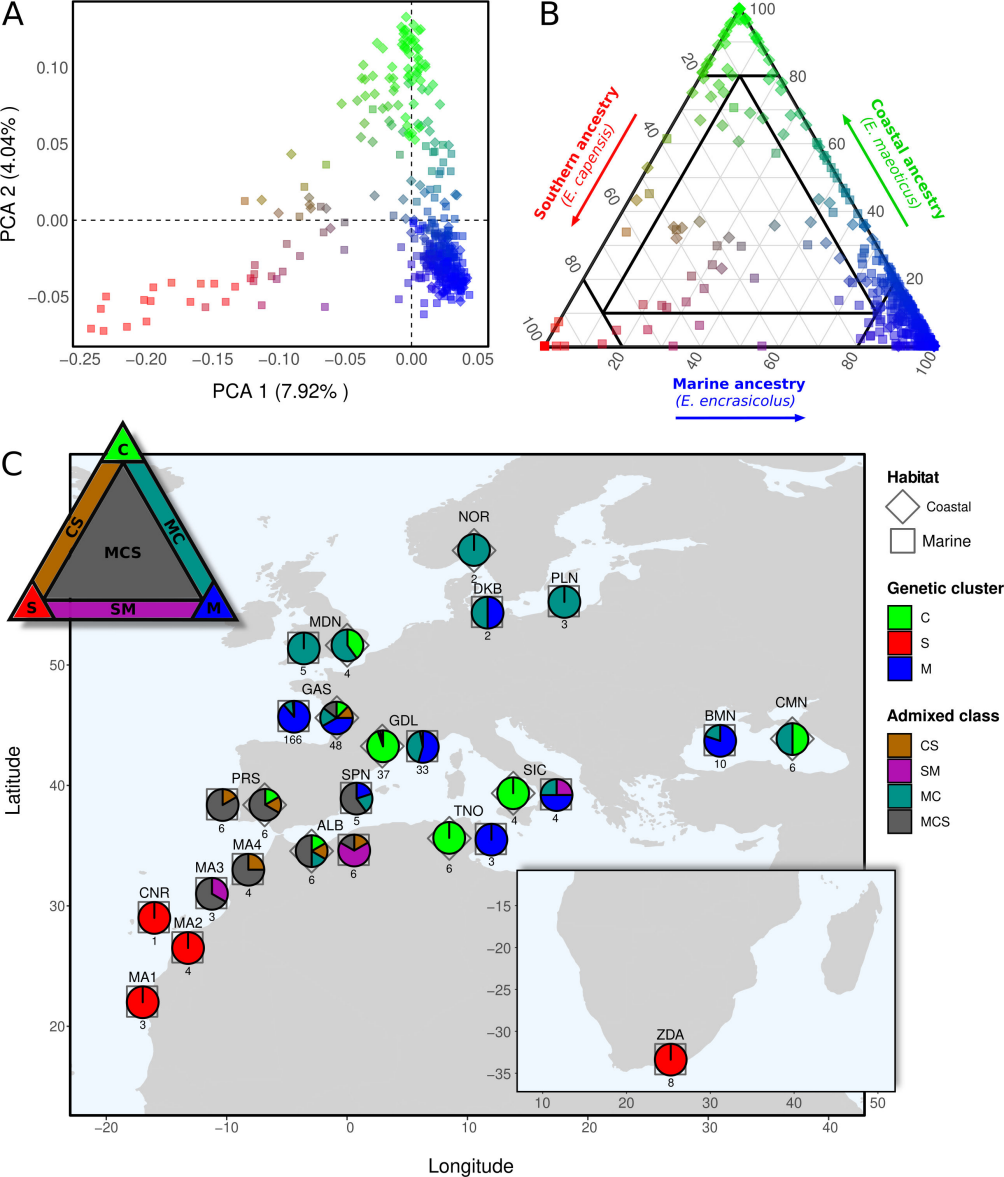
(A) Principal component analysis (PCA) performed on the entire dataset of 385 anchovy samples. Sites used in the analysis were high-quality variants present both in the WGS as well as in the RAD dataset, corresponding to a total of 3881 SNPs (MAF > 0.05). Shapes indicate habitat type and colours reflect ancestry proportions as determined by admixture analysis (see B). (B) Ternary plot showing the admixture level between three genetic ancestries: coastal (green), southern (red) and marine (blue) ancestry. Coordinates, as well as RGB colours, reflect the relative ancestry proportions of samples along each of the three axes. Samples were classified as belonging to a genetic cluster (black lines demarcating seven areas) based on their position in the plot. Clusters *C*, *S* and *M* represent parental ancestries with low admixture, while *CS*, *SM*, *MC* and *MCS* represent mixed ancestries at varying levels of admixture. (C) Map of sampling locations where symbols represent habitat type and pie charts show the proportions of different parental genetic clusters and admixed classes present. Numbers beneath pie charts indicate sample sizes.

Samples were collected from multiple sites covering a large part of the species distribution area (electronic supplementary material, table S2) and were issued from various sampling expeditions and local fisheries (electronic supplementary material, table S1). These samples were collected in different habitats, classified either as coastal or marine (symbols in figure 1C). Also included were eight individuals collected off the South African coast (Gqeberha). Whole genomic DNA was extracted from muscle tissue or fin clips using commercial tissue kits (Qiagen and Macherey-Nagel). Extraction quality was checked and standardized in concentration before library construction.

### (b) Reference genome assembly

We performed high-coverage linked-read (10× genomics) sequencing of a marine Atlantic *E. encrasicolus* individual from the Faro location (Algarve) to generate a new reference genome assembly (hereafter called Eencr_V1), following the same methodology as in Meyer et al. [30]. The *de novo* assembly obtained by analysing preprocessed linked-reads (raw coverage ~40×) with supernova v2.1.1 [31] reached a total length of ~926 Mb (925 873 119 bp; contig N50 = 13.08 kb; scaffold N50 = 20.36 kb). All downstream analyses required for variant calling from RAD-seq and WGS data were performed on the subset of scaffolds longer than 10 kb to account for assembly fragmentation. Genomic landscapes of differentiation and local PCA (see below) were reconstructed after anchoring scaffolds to the recently released chromosome-level assembly of an *E. encrasicolus* individual from the Black Sea (GenBank assembly accession: GCA_034702125.1) (electronic supplementary material, figure S1). Whole-genome alignment between Eencr_V1 and the new assembly was performed with Minimap2 [32] and visualized using D-GENIES [33].

### (c) Whole-genome resequencing data

Thirty-nine samples (electronic supplementary material, table S1) were selected for individual WGS, following the procedure described in the electronic supplementary material methods. Briefly, reads were aligned to the Eencr_V1 reference genome, and variants were called using GATK [34,35], before being filtered using vcftools v0.1.16 [36] and bcftools v1.19 [37] to retain only high-quality SNPs (see electronic supplementary material methods for filter details). The final VCF file (hereafter referred to as the *WGS dataset*) contained ~5.9 M sites located on 9093 different scaffolds longer than 10 kb.

### (d) RAD sequencing data

RAD-seq libraries were prepared for 243 samples (electronic supplementary material, table S1) following Baird *et al*. [38] (see electronic supplementary material, methods). Twenty-five of these samples were also used to produce WGS dataset, providing a link to understand the genetic structure in both datasets. To complement our sampling, we also included raw sequencing data for 128 individuals from Le Moan *et al*. [26]. Reads were aligned and processed using Stacks 2.60 for variant calling and filtering [39]. The RAD VCF was filtered to only retain sites that were present in the WGS dataset, since our objective was to describe the same genetic variation but at a larger geographic scale. Lastly, all samples from the WGS dataset were integrated into the RAD VCF. The final VCF file (hereafter referred to as the *RAD dataset*) contained genotype data for 385 samples at 3906 variable sites.

### (e) Population structure

To describe the genetic structure in both WGS and RAD datasets, we conducted genome-wide and chromosome-wide principal component analysis (PCA) using the R package SNPRelate (v1.28.0) [40]. We used ADMIXTURE v1.3.0 [41] to estimate individual ancestry proportions in all samples using the RAD dataset with default parameters. Based on results from the PCA, we assumed *K = 3* parental ancestries. Individual ancestry proportions were visualized in a triangle plot based on their ternary coordinates, and samples were then classified into different non-admixed and admixed ancestry categories based on their positions. Genetic differentiation (*F*_ST_), nucleotide diversity (*π*), absolute genetic divergence (*d*_XY_) and individual heterozygosity were calculated for the WGS data in non-overlapping 5 kb windows (with ‘--minSites 15’) using the popgenWindows.py script [42]. We also calculated the squared correlation coefficient between genotypes at SNPs (‘--geno-r2’) using vcftools v0.1.16 [36]. We tested for long-range haplotypes that are at or near fixation in one population but polymorphic in the other, using the cross-population extended haplotype homozygosity (XP-EHH) test [43] to detect signals of selection between coastal and marine ecotypes on unphased data with selscan v2.0 [44].

### (f) Identification and genotyping of structural variants

Our analyses revealed the presence of large SVs of several megabases that segregate across the distribution range (see §3). We therefore used chromosome-wide PCA to identify clusters of individuals representing alternate genotypes at each SV and assigned individuals’ genotypes based on their cluster membership using both the WGS and RAD datasets (electronic supplementary material, figure S2). The PCA axis representing structural variation was evidenced by the presence of three separated clusters and was in most cases supported by PC1. Samples which did not show clear cluster membership based on their PCA coordinates were not genotyped. For samples that had WGS data, we corroborated genotype assignment with the relative positions of each sample in local PCA, which was conducted in non-overlapping windows of 5 kb using lostruct (v0.0.0.9L) [45]. In total, we genotyped individuals at 13 large SVs that occur on different chromosomes.

### (g) Divergence history of structural variants

To study the evolutionary relationships between individuals carrying different haplotypes at the 13 SVs, we constructed neighbour-joining phylogenetic trees for each chromosome. Trees were obtained using the ‘phylo’ command from VCF-Kit [46], which uses variable sites from a VCF to create a multiple sequence alignment to then calculate a difference matrix using MUSCLE [47]. Because our chromosome-wide PCAs indicated low recombination between alternate SV haplotypes on the 13 chromosomes, these SV trees can be used to resolve the evolutionary relationships among haplotypes without the confounding effect of recombination. For these analyses, we used a subset of high-coverage individuals that are homozygous for the SV to avoid phasing issues and to facilitate visualization of haplotype relationships. We rooted trees on the branch separating alternate homozygote groups. For comparison, trees were also constructed for chromosomes not carrying SVs and rooted using a South African sample (‘ATL_MAR_ZDA_61_1162’).

## 3. Results

### (a) Genetic structure in the *E. encrasicolus* species complex reveals three-way admixture

We combined WGS and RAD-seq data to study the genetic structure of anchovies in the eastern Atlantic Ocean, Mediterranean Sea and Black Sea. The RAD dataset, with mean per-sample coverage of 52.7X (mapping results provided in the supplied HTML report, see electronic supplementary material, appendix), revealed a clear picture of the overall genetic structure in the *E. encrasicolus* species complex across its entire range distribution. These results from a reduced representation SNP dataset (3906 SNPs) closely matched those obtained using our WGS dataset (5.9 M SNPs, per-sample coverage 10−30X), which contains a smaller subset of samples (see WGS PCA in the electronic supplementary material, appendix). Firstly, we observed genetic differentiation between samples collected in marine and coastal habitats in the northern part of the range, corresponding to the previously described marine and coastal ecotypes [26,27]. This can be observed along the second axis of variation (PCA 2 in figure 1A), whereas PCA 1 shows a different signal that reflects geographic structure rather than ecological structure. On this horizontal axis, South African samples and other individuals collected off the African Atlantic coast (Morocco and the Canary Islands) are spread out towards the left-hand side of the plot, while the majority of other samples group to the right. Hence, PCA at a genome-wide scale shows the existence of three distinct genetic ancestries, which were further confirmed using admixture analysis. Individual ancestry was represented in a ternary plot (figure 1B) showing the relative proportions of coastal (top), marine (right) and southern (left) ancestry for each individual. Ongoing gene flow between the three ancestries was revealed by substantial levels of admixture, in particular between the marine and coastal ancestries. A large number of samples also fell in the central area of the plot due to balanced proportions of the three ancestry components, reflecting the existence of three-way admixture.

Based on their ternary coordinates, samples were grouped into seven ancestry categories, each corresponding to a defined sub-area of the triangle plot (black demarcations figure 1B, triangle in figure 1C). A sample was thus considered to have a dominant ancestry contribution from a genetic cluster (coastal/*C*: green; southern/*S*: red; marine/*M*: blue) if that ancestry accounted for over 80% of its total genetic makeup. We distinguished three admixed classes where two ancestries dominated (and the third did not amount to more than 10%): *CS* (admixed between *C* and *S*; brown), *SM* (admixed between *S* and *M*; purple) and *MC* (admixed between *M* and *C*; seagreen). A last admixed class, called *MCS*, consisted of individuals with balanced proportions of all three ancestries (admixed between *M*, *C* and *S*; grey). The eco-geographical distribution of the three parental ancestries revealed that individuals belonging to the *C* cluster (green) were only found in coastal habitats (diamond symbols) in the northern part of the range, while *M* individuals (blue) mainly occurred in marine environments (square symbols) of the same region (figure 1C). This corresponds to the previously described ecotypic structure between coastal and marine anchovies, which is especially pronounced in the Mediterranean Sea. However, this signal of ecotypic differentiation becomes diluted near the Atlantic–Mediterranean boundary, where a gradient of increasing southern ancestry is observed. This admixture gradient can be seen through the increasing proportion of *MCS* individuals (grey) in the Alboran Sea (*ALB*), off the southern coast of Portugal (*PRS*) and in northern Morocco (MA4 and MA3). Finally, we observed that samples from locations to the south of the Canary Islands (*CNR*), including South Africa (*ZDA*, inset map), all belonged to the *S* cluster (red) and were found in marine habitats.

**Figure 2.**
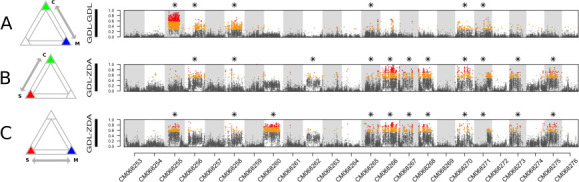
Genomic landscapes of differentiation (*F*_ST_) were calculated in 5 kb sliding windows between groups of samples (3 individuals per group) from different genetic clusters. Differentiation landscapes are shown for three different comparisons (A: coastal versus marine; B: coastal vs. southern; C: southern vs. marine), where all marine and coastal samples are from Mediterranean locations (‘GDL’). Orange points are windows where *F*_ST_ was higher than the 95^th^ quantile, while red points are above the 99th quantile. Stars indicate chromosomes where more than 2.5% of windows showed *F*_ST_ higher than the 95^th^ quantile. Grey and white rectangles delimit the 24 chromosomes of *E. encrasicolus*.

After describing the three ancestries as well as their ecogeographic distribution patterns, we aimed to study their genomic architecture of differentiation. *F*_ST_ landscapes reconstructed between the coastal, marine and southern clusters using WGS data, yielded highly heterogeneous patterns that strongly varied from chromosome to chromosome (figure 2). The background level of differentiation between the marine and coastal clusters was lower (figure 2A, mean *F*_ST_ = 0.013) compared to the background *F*_ST_ between the southern and coastal clusters (figure 2B, mean *F*_ST_ = 0.075) and between the southern and marine clusters (figure 2C, mean *F*_ST_ = 0.066) (electronic supplementary material, figure S12). *F*_ST_ landscapes between marine and coastal individuals in the Mediterranean Sea (figure 2A) were similar to landscapes in the Atlantic (electronic supplementary material, figure S3), even though some differences were observed (e.g. on chromosomes CM068259 and CM068273).

Genomic differentiation landscapes were largely characterized by the presence of high-differentiation regions (*F*_ST_ values above the 95^th^ quantile) clustering into continuous plateaus. These patterns could point to the presence of large SVs associated with ecotype or lineage divergence. We thus aimed to assess whether these putative SVs comprise blocks of SNPs that are in strong LD and whether these regions harbour divergent, non-recombining haplotypes. The mean squared correlation coefficient (*R* ^2^) between windows of consecutive SNPs revealed tight LD regions coinciding with *F*_ST_ peaks on several chromosomes (electronic supplementary material, figure S4). Additionally, local PCAs on individual chromosomes detected tight clusters of samples that were consistent between the WGS and RAD datasets (electronic supplementary material, figures S5 and S7) and showed continuous clustering of genotypes across numerous consecutive windows (electronic supplementary material, figure S2), with elevated levels of heterozygosity in the intermediate clusters (electronic supplementary material, figure S14). These results indicate that groups of SNPs in strong LD result in the segregation of a limited number of non-recombining haplotypes, providing further support for the presence of multiple large SVs in the anchovy genome.

While some chromosomes exhibited several discrete clusters of tightly grouped samples, others showed continuous ancestry gradients and lower variation explained by the first two PC axes. In total, we identified 13 chromosomes (indicated by asterisks in figure 2) with evidence of SVs spanning at least 2.5% of the windows on the chromosome. Using individual coordinates from chromosome-wide PCAs, these 13 SVs were genotyped (electronic supplementary material, figures S6 and S8), classifying individuals as either *00* homokaryotes (pink), *01* heterokaryotes (salmon) or *11* homokaryotes (gold). Individuals that could not be confidently assigned to any given group were not genotyped (grey). We always assigned the *00* genotype to the group with the most southern samples, polarizing the *0* haplotype with respect to southern ancestry. For the SV located on chromosome CM068256, the PCAs based on the WGS and RAD datasets showed different results (electronic supplementary material, figures S5 and S7), which complicated our assignment of genotypes for this SV. We opted to genotype this SV according to variation captured by PC1 in the WGS analysis, which included more markers and matched structure in the phylogenetic reconstruction (electronic supplementary material, figure S10).

### (b) Anchovy lineages are differentiated at multiple SVs

**Figure 3.**
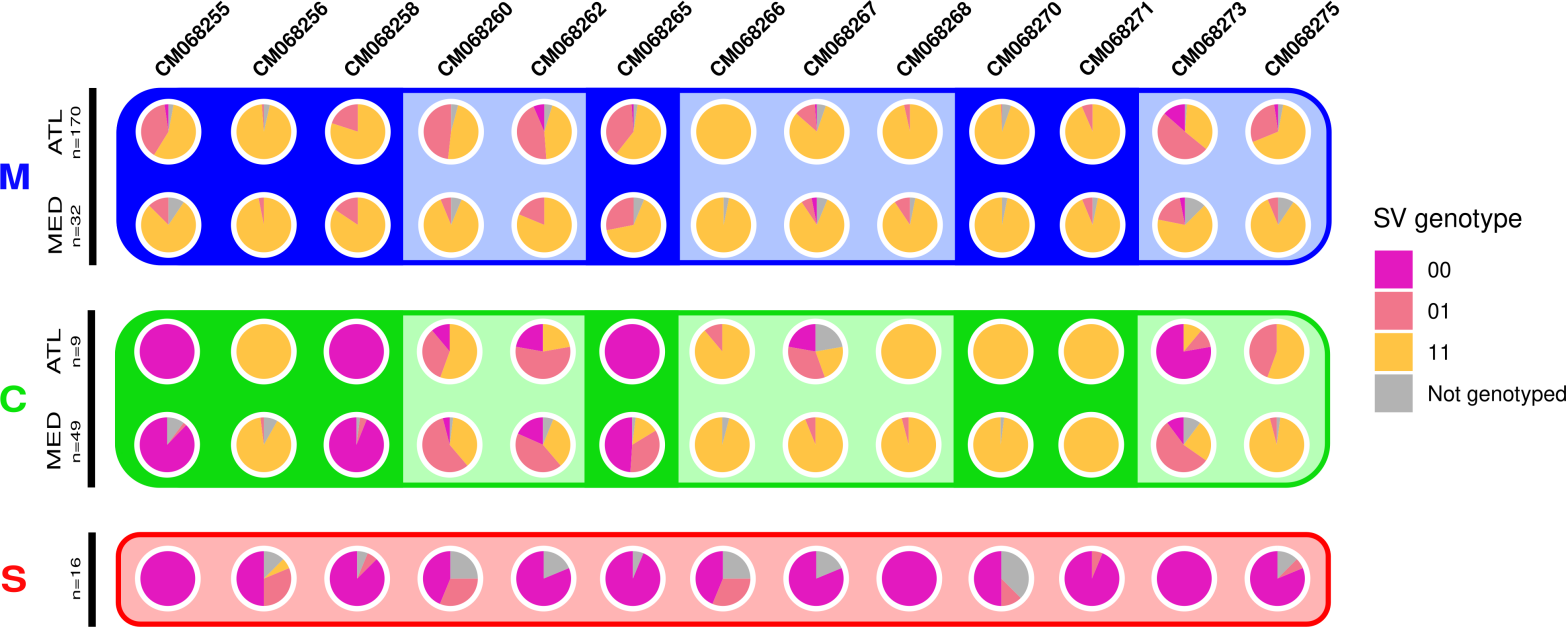
Genotype frequencies for 13 SVs on different chromosomes (columns). Pie charts show frequencies for the *00* (pink), *01* (salmon) and *11* (gold) genotypes in the marine cluster (blue background), coastal cluster (green background) and southern cluster (red background), with upper and lower rows corresponding to Atlantic and Mediterranean samples, respectively (for *M* and *C*). Darker background colour in *M* and *C* indicates chromosomes that show elevated *F*_ST_ when comparing marine and coastal individuals (figure 2A). For three of these chromosomes (CM068255, CM068258 and CM068265), ecotype differentiation involves southern haplotypes (*00*) that are present at high frequency in the coastal individuals, while this is not the case for CM068256, CM068270 and CM068271.

Based on assigned SV genotypes, we analysed haplotype frequency patterns (*0* and *1*) (electronic supplementary material, figure S11) as well as genotype frequencies (*00*, *01* or *11*) within each genetic cluster (figure 3). Coastal, marine and southern clusters (background colours in figure 3) carried distinct sets of SV genotypes, consistent with the *F*_ST_ plateaus observed in differentiation landscapes (figure 2). The southern cluster (bottom row of pie charts) largely harboured homokaryotic *00* genotypes (mean *00* frequency of 77% across SVs, 6 out of 13 fixed), while marine individuals (top two rows) were predominantly homokaryotic *11* (mean *11* frequency of 79% across SVs). By contrast, in coastal individuals, certain SVs were dominated by *00* and others by *11* genotypes (see details below). Admixed individuals (e.g. *MCS*) were often heterokaryotes at SVs (electronic supplementary material, figure S9), reflecting their mixed ancestry.

Although SV frequencies differ among the coastal, marine and southern clusters, these SVs are not always fixed for a given haplotype but often display a degree of haplotype sharing. This is clearly visible on CM068262 and CM068273, for example, where the SVs are polymorphic in almost all the northern populations (figure 3). By contrast, our XP-EHH analysis between coastal and marine ecotypes revealed some chromosomes with long-range haplotypes nearly fixed in one population but polymorphic in the other (e.g. CM068255, CM068258, CM068271; electronic supplementary material, figure S15).

**Figure 4.**
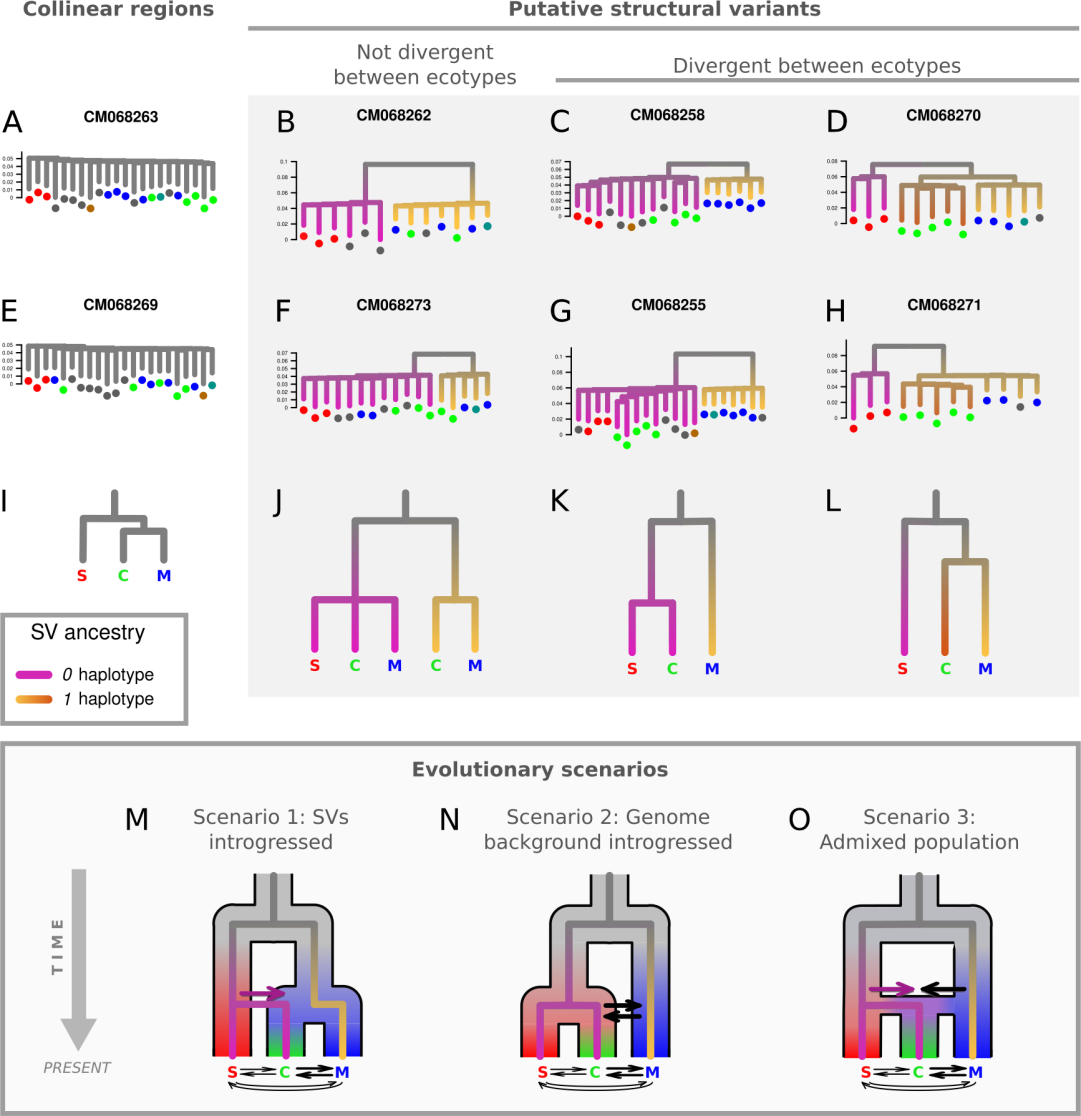
(A−H) Neighbour-joining trees showing interindividual relationships on a subset of chromosomes. Short branch lengths for normally recombining chromosomes (A,E) contrast with long branches between divergent haplotypes at SVs (B−D,F−H). These SVs show varying patterns of being either shared or private to the coastal, marine or southern clusters, as is illustrated with schematic trees in (I−L) where pink or gold coloration reflects divergence between haplotypes. In (L), brown coloration indicates the divergence of a third haplotype differentiating coastal and marine samples (e.g. CM068270 and CM068271). We propose three evolutionary scenarios (M–O) involving gene flow among lineages (horizontal arrows) which could explain the observed patterns of haplotype distributions across SVs. For (A,E), trees were constructed using SNPs on the entire chromosome, whereas in (B−D,F−H), the region was limited to that covered by the SV. For trees of SV regions, intermediate samples that present heterokaryotes are not displayed. Leaf tips are coloured according to individual ancestry categories. Trees were plotted on the same vertical scale.

We found that southern ancestry haplotypes (*0*) are common in marine and/or coastal populations in the north. These southern haplotypes are slightly more common in the Atlantic (first and third rows in figure 3) than in the Mediterranean (second and fourth rows). A key finding is the substantial excess of haplotype sharing at SVs between the southern and coastal clusters (mean *F*_ST_ = 0.218 in SV regions), compared to the southern and marine clusters (mean *F*_ST_ = 0.273 in SV regions). This is particularly clear on CM068255, CM068258 and CM068265, where *0* haplotypes predominate or are fixed in the coastal samples. These three chromosomes are among the six with high *F*_ST_ between the marine and coastal clusters (asterisks in figure 2A), probably due to the presence of southern haplotypes in coastal samples. This was confirmed in phylogenies of the SV regions (figure 4C,G), since coastal and southern samples are grouped in the same branch (pink), while marine samples (alternative haplotype) form a separate branch (gold).

On the other hand, high *F*_ST_ between coastal and marine samples on CM068256, CM068270 and CM068271 is not explained by southern haplotypes. On these chromosomes, northern populations almost exclusively carried *1* haplotypes (rows 1−4 in figure 3) that were not only distinct from the *0* haplotype, but also divergent between *C* and *M*. The presence of haplotype structure within the *1* haplogroup is indeed suggested by the separation of coastal and marine samples on PCA 2 (electronic supplementary material, figure S5), subdivision between different *1* haplotypes in phylogenies (brown and gold branches in figure 4D,H), and extreme XP-EHH values (electronic supplementary material, figure S15). Other SVs also showed evidence for three distinct haplotypes (e.g. CM068273), but only CM068256, CM068270 and CM068271 were investigated (haplotype frequencies shown in electronic supplementary material, figure S11) as they are involved in ecotype differentiation (figure 2A). Overall, these patterns suggest that there are three distinct haplotypes segregating on CM068256, CM068270 and CM068271, potentially resulting from multiple SVs on each chromosome.

We studied the distribution of *d*_XY_ values (electronic supplementary material, figure S13) and branch lengths separating haplotypes in phylogenies (electronic supplementary material, figure S10) to assess divergence in different genomic regions. We observed different types of patterns that are illustrated by the four columns in figure 4. Chromosomes without large SVs are characterized by short branch lengths and low levels of divergence (figure 4A,E). These collinear regions did not show pronounced genetic structure and supported the highest similarity between the coastal and marine clusters. These patterns contrasted with phylogenies reconstructed from SV regions, where long branches were found to separate samples carrying different genotypes (figure 4B–D,F–H). Distributions of *d*_XY_ values between alternate homokaryotes were relatively similar across SVs, with mean *d*_XY_ between 0.15% and 0.20%. Divergence between coastal *1* haplotypes and marine *1* haplotypes on CM068256, CM068270 and CM068271 was lower, as is reflected by shorter branches in phylogenies (figure 4D,H). However, SV block delimitation was probably not precise enough to exclude all recombinant regions and to provide precise divergence estimates.

## 4. Discussion

We present the first genome-scale study of the *E. encrasicolus* species complex, comprising anchovies from the eastern Atlantic Ocean, Mediterranean Ocean and Black Sea. Our findings reveal that two previously described ecotype lineages (marine and coastal) genetically interact with a third, southern lineage. This lineage has not previously been described as such, even though some anchovy samples from the northeast Atlantic and from southern Morocco were shown to display genetic proximity with *E. capensis* [28,29]. Although taxonomic considerations are beyond the scope of this study, our results show that the coastal, marine and southern lineages are primarily differentiated by genotype combinations at multiple large SVs, with the collinear parts of their genomes being weakly differentiated. We further found that these SVs are most likely to have originated from admixture with the southern lineage, and that the coastal ecotype carries southern haplotypes at several SVs. Our study supports that SVs that arose in geographically isolated lineages can play an important role in driving ecotype formation following lineage admixture.

Previous genetic studies have shown that the European anchovy is subdivided into marine and coastal ecotypes that are present from the Bay of Biscay, through the Mediterranean to the Black Sea (reviewed in [27]). Here, we show that there is a third genetic component in this species complex, corresponding to an Atlantic lineage occurring off the African coastline. This southern lineage shows genetic homogeneity at a very large spatial scale, with genetic similarity between individuals sampled in Morocco, the Canary Islands and even as far as South Africa. However, from northern Morocco and southern Portugal into the Alboran Sea, we observe genetic admixture resulting in a gradient of decreasing southern ancestry. Previous studies reporting various signals of spatial structure in this species may have unknowingly captured different aspects of these complex admixed ancestries, leading to many different and conflicting interpretations in the literature. For instance, Zarraonaindia et al. [29] reported the presence of an admixture gradient extending from the Canary Islands to the northern Iberian peninsula. However, this gradient was attributed to the interaction of two components corresponding to populations inhabiting narrow versus wide shelf waters. We instead propose that this region corresponds to a three-way contact zone between the southern lineage and the two northern marine and coastal ecotype lineages, thus involving both an ecological component as well as admixture between geographic lineages. We observe post-F1 introgressive hybridization resulting in widespread admixture and gene flow between the southern, coastal and marine genetic clusters, as is reflected by gradual ancestry gradients in the PCA plot (figure 1). The existence of gene flow between coastal and marine ecotypes has already been illustrated in previous work [26], but our results reveal that admixture with the southern lineage also contributes to global diversity patterns.

We found evidence for multiple megabase-scale SVs that segregate in the marine, coastal and southern anchovy lineages. Although the presence of SVs was only supported through indirect evidence (LD, divergence and heterozygosity patterns), these regions showed many of the signals typically associated with inversions [48]. SVs are frequently found to play a role in differentiating evolutionary lineages or promoting ecotype formation [16,49]. Our results in anchovies seem to point in this direction, since markers differentiating lineages and ecotypes were largely found in SVs, whereas collinear regions of the genome showed low differentiation due to gene flow and recombination. By reconstructing the genomic landscape of ecotype divergence, we identified six large SVs that differentiate marine and coastal ecotypes. These SVs collectively cover roughly 25% of the genome, which is in line with a previous study estimating that the barriers to gene flow between ecotypes span 20–25% of the genome [26].

Our results also revealed that the origin of the six SVs differentiating ecotypes is likely to be a consequence of admixture between divergent geographic lineages. In particular, the coastal ecotype shares the same haplotype as the southern lineage at a minimum of three SVs. If these SVs were already segregating in the population ancestral to all three lineages, different conflicting genealogies would have been expected due to incomplete lineage sorting (ILS). However, we do not observe any SVs where the marine and southern lineages share a haplotype that is divergent from the coastal ecotype. Overall, the patterns of population structure in the collinear genome, along with haplotype distributions at SVs (whether shared or lineage-specific) suggest three alternative scenarios that could explain the observed genealogical patterns (figure 4M–O):

(1) In the first scenario, SVs could have been introgressed from the southern lineage into the coastal lineage (figure 4M). The deepest split marks the initial divergence between the southern lineage and the northern ancestral branch giving rise to the marine and coastal ecotypes. In this scenario, southern haplotypes may have been introgressed into a pre-existing coastal lineage or may have contributed to the formation of the coastal ecotype. This introgression probably occurred during an earlier period of contact, since current admixture in the contact zone seems insufficient to explain why coastal samples from across the distribution range almost exclusively carry southern haplotypes at these SVs.
(2) The second scenario proposes that the coastal ecotype directly originated from the southern lineage and does not share its most recent common ancestor with the marine lineage (figure 4N). In this case, recent common ancestry between the coastal and southern lineages would only be observed at the SVs, as widespread introgression with the marine lineage has eroded divergence in the collinear genome. This scenario does not require multiple SVs to pass through various selective filters to establish. Instead, it involves neutral introgression in the background genome, as well as polymorphism at certain SVs shared via gene flow.
(3) Another alternative scenario is genome-wide admixture, where the coastal ecotype originated from an admixed population composed of both southern and northern ancestries (figure 4O). This admixture could have been followed by selective reassortment of SVs, driven either by adaptation to the coastal environment [7] or by the resolution of genomic conflicts [21].

A previous demographic inference study suggested that the marine and coastal ecotypes experienced secondary contact after divergence in allopatry [26]. Including a third anchovy lineage in such analyses, while explicitly accounting for SVs, would improve our understanding of admixture and introgression events that shaped ecotype formation. However, this would require specific methodological developments beyond the scope of the present study. Nonetheless, the period of allopatric divergence previously inferred in Le Moan et al. [26] may actually correspond to the split between northern and southern lineages (figure 4M–O). This divergence signal may have been preserved within the SVs, while subsequent gene flow and recombination eroded it in the background genome.

In addition to the six SVs that separate coastal and marine anchovies, we also identified other SVs (e.g. on CM068260) that are shared between ecotypes and where one haplotype has a southern lineage origin. Here again, the lack of recombination between rearrangements has preserved the historical divergence signal, despite these SVs being exchanged between ecotypes. This result closely mirrors what has been described by studies using mitochondrial data, where two deeply divergent clades (called A and B) were found to segregate in populations now known to correspond to the marine and coastal ecotypes [50,51] and the southern lineage [28,52–54]. Therefore, the mitochondrial genome and the shared SVs probably represent genomic regions that have retained the signal of north–south divergence despite gene flow after secondary contact.

The last type of genealogical pattern that we identified separates the southern lineage from coastal and marine ecotypes that share more recent common ancestry (figure 4D,H), displaying the same topology as the collinear genome but with much longer branches. This suggests that, in addition to north–south divergence, there is also more recent divergence between the two northern ecotypes, possibly involving complex rearrangements such as nested inversions [55,56]. Alternatively, these SVs could have a similar evolutionary history as proposed above in scenarios 1, 2 and 3. Namely, coastal anchovy would have carried a southern haplotype in the past, before it recombined with the northern haplotype due to gene flow between ecotypes, consequently decreasing the level of divergence between haplotypes. Gene flow between inversion arrangements has been described in other systems, in particular for large chromosomal inversions that are impacted by gene conversion and double crossovers [30,57,58].

Overall, we found that secondary contact between northern and southern anchovy lineages—whether involving SV introgression (scenario 1), background genome introgression (scenario 2), or genome-wide admixture (scenario 3)—is sufficient to explain the diversity of genealogical patterns observed. However, our results do not allow us to determine which of the three alternative evolutionary scenarios is the most likely. This would require more in-depth demographic reconstruction and complementary information regarding past geographic distributions, particularly during the last glacial period. We may hypothesize that north–south divergence probably took place across hemispheres, as it broadly reflects current lineage distributions. Alternatively, divergence may have occurred between the northeast and northwest Atlantic, since populations from the Gulf of Mexico may also belong to the *E. encrasicolus* species complex [28,54]. Multiple periods of secondary contact could have occurred during cooler periods, when long-distance range shifts were possible and lineages were confined to lower latitudes due to polar ice sheets [59,60].

It remains unresolved, however, whether ancestral anchovy lineages were associated with specific habitats or how/when this association arose to result in coastal and marine ecotypes. Today, the southern lineage seems to be associated with marine habitats along the Moroccan, Mauritanian, Namibian and South African coasts [61,62]. In this light, it is not obvious how the southern SVs haplotypes, carried by the coastal ecotype, could underlie adaptation to coastal environments. However, it is possible that epistatic interactions with loci in the genomic background have affected ecological traits that were not initially associated with these haplotypes. Alternatively, mutations could have accumulated in the SVs over time [63], conferring local adaptation to the coastal environment that was absent in the southern lineage.

Our study highlights the crucial role of SVs and admixture in the formation of ecotypes, supporting that hybridization between geographically isolated lineages can provide the genetic substrate necessary for ecological specialization and partial RI. SVs can act as ‘pre-packaged’ divergent blocks containing multiple genes that contribute to RI through local adaptation, coadapted gene complexes or incompatibilities. This supports a broader view that ecotype formation is rarely driven by *in situ* adaptation alone, but often involves phases of allopatric divergence and other historical contingencies [ 6,64–66]. After admixture, SVs can fuel ecotype differentiation and resist gene flow over the long term, facilitating coupling among RI components. These associations are necessary to maintain ecotypes and are instrumental if speciation is ever to complete [67,68]. Coupled SVs may be especially important for maintaining differentiation in high gene flow marine species [69–71]. However, whether SVs alone are sufficient for marine ecotypes to speciate remains uncertain [72] and depends on the moving balance between gene flow and divergent selection. Future research should explore the broader role of admixture as a source of divergent SVs and trace their evolutionary history to reveal how these variants contribute to the formation of ecotypes across taxa.

## Data Availability

All sequencing data have been deposited in the NCBI GenBank Sequence Read Archive. RAD-seq reads are available under BioProject ID PRJNA311981, with sample accessions from SAMN48731832 to SAMN48732074. These can be accessed at https://www.ncbi.nlm.nih.gov/biosample?LinkName=bioproject_biosample_all&from_uid=311981. Whole-genome sequencing (WGS) reads are available under BioProject ID PRJNA777424, with sample accessions SAMN48800104 to SAMN48800125 and SAMN48746249 to SAMN48746268. These can be accessed at https://www.ncbi.nlm.nih.gov/biosample?LinkName=bioproject_biosample_all&from_uid=777424. VCF files and an HTML-based supplementary appendix, containing detailed methods and outputs, are available via Dryad [73]. All code used for data processing and analysis is included in the main text or electronic supplementary material [74].
